# 2-Chloro-6-[(2,4-dimeth­oxy­benz­yl)amino]-9-isopropyl-9*H*-purine

**DOI:** 10.1107/S1600536813004121

**Published:** 2013-02-16

**Authors:** Radka Novotná, Zdeněk Trávníček

**Affiliations:** a Department of Inorganic Chemistry, Faculty of Science, Palacký University, 17. listopadu 12, CZ-771 46 Olomouc, Czech Republic

## Abstract

In the title compound, C_17_H_20_ClN_5_O_2_, the benzene ring and the purine ring system make a dihedral angle of 78.56 (4)°. In the crystal, mol­ecules are linked by pairs of N—H⋯N hydrogen bonds, forming inversion dimers. C—H⋯O and C—H⋯Cl contacts further link the mol­ecules, forming a three-dimensional network.

## Related literature

For the synthesis, see: Oh *et al.* (1999 ▶). For related structures, see: Trávníček & Popa (2007*a* ▶,*b* ▶); Trávníček *et al.* (2010 ▶); Čajan & Trávníček (2011 ▶). For the cytotoxic activity of related compounds, see: Benson *et al.* (2005 ▶); Meijer *et al.* (1997 ▶); Štarha *et al.* (2010 ▶); Vrzal *et al.* (2010 ▶).
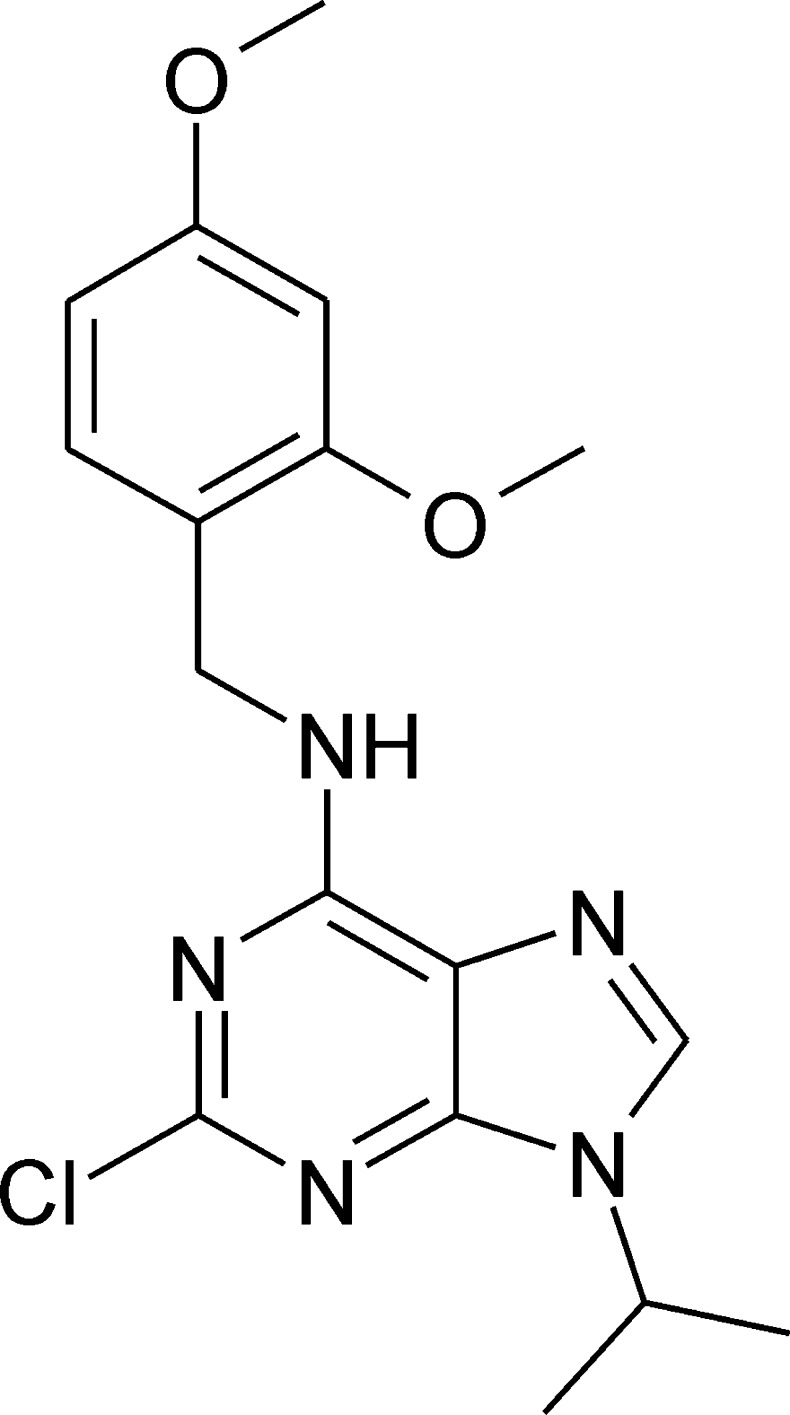


## Experimental

### 

#### Crystal data

C_17_H_20_ClN_5_O_2_
*M* *_r_* = 361.83
Triclinic, 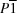

*a* = 7.8620 (2) Å
*b* = 9.20164 (18) Å
*c* = 13.3027 (3) Å
α = 82.4472 (18)°
β = 74.803 (2)°
γ = 66.012 (2)°
*V* = 848.16 (3) Å^3^
*Z* = 2
Mo *K*α radiation
μ = 0.25 mm^−1^
*T* = 100 K
0.40 × 0.35 × 0.30 mm

#### Data collection

Agilent Xcalibur Sapphire2 diffractometer
Absorption correction: multi-scan (*CrysAlis PRO*; Agilent, 2012 ▶) *T*_min_ = 0.908, *T*_max_ = 0.930
7228 measured reflections
2965 independent reflections
2704 reflections with *I* > 2σ(*I*)
*R*_int_ = 0.009

#### Refinement

*R*[*F* ^2^ > 2σ(*F* ^2^)] = 0.029
*wR*(*F* ^2^) = 0.079
*S* = 1.10
2965 reflections
230 parameters
H-atom parameters constrained
Δρ_max_ = 0.28 e Å^−3^
Δρ_min_ = −0.19 e Å^−3^

### 

Data collection: *CrysAlis PRO* (Agilent, 2012 ▶); cell refinement: *CrysAlis PRO*; data reduction: *CrysAlis PRO*; program(s) used to solve structure: *SHELXS97* (Sheldrick, 2008 ▶); program(s) used to refine structure: *SHELXL97* (Sheldrick, 2008 ▶); molecular graphics: *Mercury* (Macrae *et al.*, 2006 ▶) and *DIAMOND* (Brandenburg, 2011 ▶); software used to prepare material for publication: *publCIF* (Westrip, 2010 ▶).

## Supplementary Material

Crystal structure: contains datablock(s) I, global. DOI: 10.1107/S1600536813004121/ng5316sup1.cif

Structure factors: contains datablock(s) I. DOI: 10.1107/S1600536813004121/ng5316Isup2.hkl

Supplementary material file. DOI: 10.1107/S1600536813004121/ng5316Isup3.cml

Additional supplementary materials:  crystallographic information; 3D view; checkCIF report

## Figures and Tables

**Table 1:** Hydrogen-bond geometry (Å, °)

*D*—H⋯*A*	*D*—H	H⋯*A*	*D*⋯*A*	*D*—H⋯*A*
N6—H6⋯N7^i^	0.88	2.16	2.9465 (15)	148
C16—H16*C*⋯Cl1^ii^	0.98	2.78	3.4607 (15)	127
C19—H19*A*⋯O2^iii^	0.98	2.57	3.4709 (17)	154

Symmetry codes: (i) 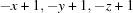; (ii) 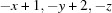; (iii) 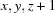.

**Fig. 1.**
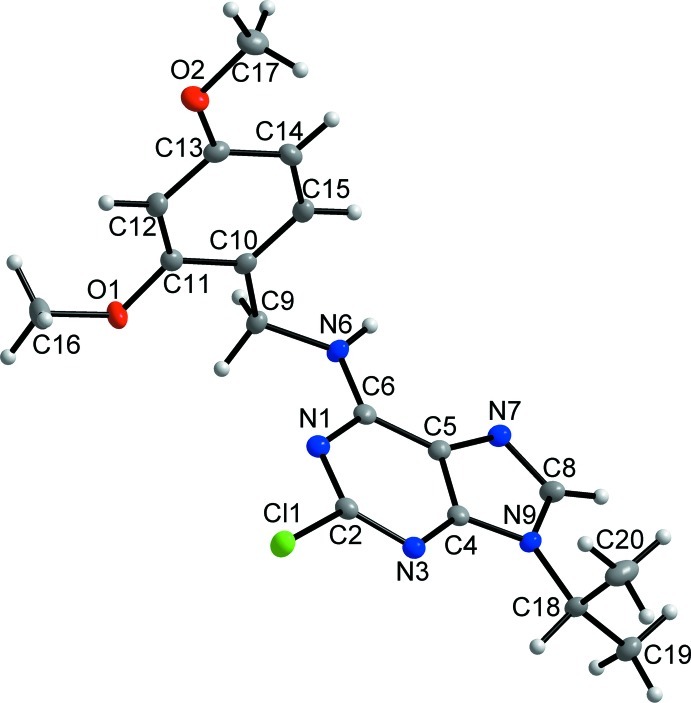
The molecular structure of the title compound (I) with the non-hydrogen atoms depicted as thermal ellipsoids at the 50% probability level shown with the atom numbering.

**Fig. 2.**
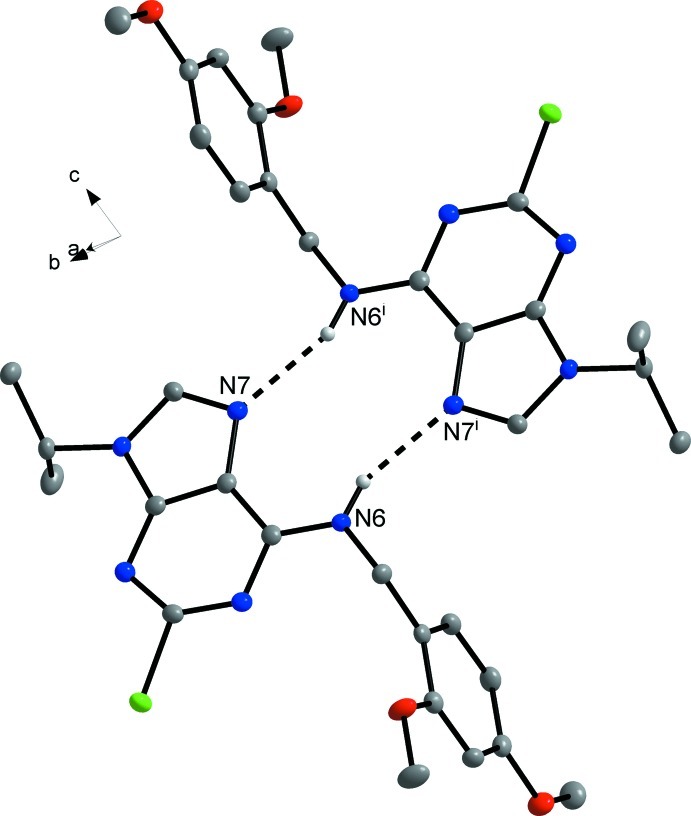
A part of the crystal structure of (I) showing the N—H···N hydrogen bonds connecting the individual molecules into centrosymmetric dimers (symmetry code: (i) -x + 1, -y + 1, -z + 1). Hydrogen atoms not involved in the contacts were omitted for clarity.

**Fig. 3.**
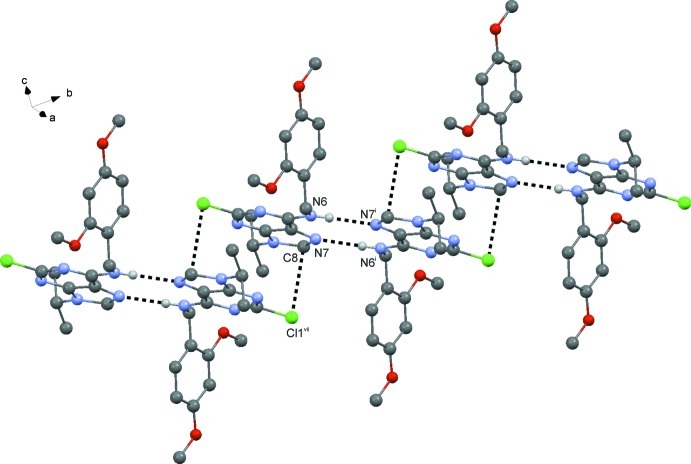
A part of the crystal structure of (I) showing the centrosymmetric dimers connected by C···Cl non-covalent contacts (symmetry codes: (i) -x + 1, -y + 1, -z + 1; (vii) 1 - x, 2 - y, 1 - z). Hydrogen atoms not involved in the contacts were omitted for clarity.

**Fig. 4.**
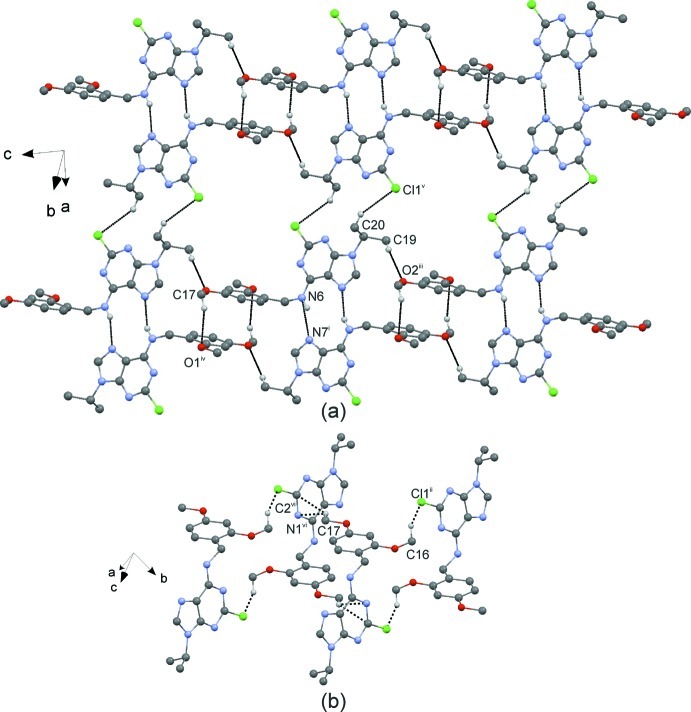
A part of the crystal structure of (I) showing the present non-covalent interactions of the C—H···O, C—H···Cl, C—H···N, C—H···C types (symmetry codes: (i) 1 - x, 1 - y, 1 - z; (ii) 1 - x, 2 - y, -z; (iii) x, y, 1 + z; (iv) 1 - x, 1 - y, -z; (v) 2 - x, 2 - y, 1 - z; (vi) 2 - x, 1 - y, -z). Hydrogen atoms not involved in the contacts were omitted for clarity.

## supplementary crystallographic information

### Comment

The title compound (I) is derived from 6-benzylaminopurine, which is along with its derivatives classified among plant growth hormones called cytokinins, which influence crucial biochemical processes, such as cell cycle and division, in plant tissues. Suitable substitutions on the 6-benzylaminopurine skeleton, particularly in the C2 and N9 positions, lead to the formation of compounds that can act as inhibitors of enzymes cyclin dependent kinases in various phases of the human cell cycle (Meijer *et al.*, 1997). One representative of such derivatives, (*R*)-Roscovitine, *i.e.* 2-[(*R*)-(1-ethyl-2-hydroxyethylamino)]-6-(benzylamino)-9-isopropylpurine (Seliciclib or CYC202), has entered the IIb phase of clinical trials in patients with nonsmall cell lung cancer (Benson *et al.*, 2005). Moreover, 6-benzylaminopurine derivatives have been successfully used as N-donor ligands in transition metal complexes exhibiting varied types of biological activity. Even the title compound (I) has been employed as a ligand in the preparation of highly anticancer active platinum(II) oxalato complexes whose *in vitro* cytotoxic activity against various human cancer cells exceeded the commercially applied drug cisplatin (Štarha *et al.*, 2010; Vrzal *et al.*, 2010).

The molecular structure of (I) consists of discrete molecules of a three-substituted adenine derivative, 2-chloro-6-[(2,4-dimethoxybenzyl)amino]-9-isopropylpurine (Fig. 1). It is basically derived from the 6-benzylaminopurine skeleton by substitutions of H atoms by the methoxy groups in the positions 2 and 4 on the benzene ring, by chlorine, and the isopropyl group in the position C2, and N9, of purine, respectively. The molecule contains two heterocyclic rings pyrimidine and imidazole, which are almost coplanar, since they form a dihedral angle of 2.02 (5)°. Additionally, there is also a benzene ring present in the structure of (I) which is similarly to the other two rings essentially planar. The maximum deviations from the least-square planes fitted through the non-hydrogen atoms for each of the rings are as follows: 0.0102 (14) Å for C4 in pyrimidine, 0.0025 (14) Å for C8 in imidazole and 0.007 (2) Å for C14 in benzene. The benzene and purine ring systems form a dihedral angle of 78.56 (4)°.

The crystal structure of (I) consists of the molecules of 2-chloro-6-[(2,4-dimethoxybenzyl)amino]-9-isopropylpurine organized into centrosymmetric dimers connected by N—H···N hydrogen bonds (Table 1, Fig. 2). Additionally, varied types of non-covalent contacts are present in the structure of (I), namely C—H···O [*d*(C19···O2^iii^) = 3.471 (2) Å; *d*(C17···O1^iv^) = 3.358 (2) Å; symmetry codes: (iii) *x*, *y*, 1 + *z*; (iv) 1 - *x*, 1 - *y*, -*z*], C—H···Cl [*d*(C16···Cl1^ii^) = 3.820 (2) Å; *d*(C20···Cl1^v^) = 3.461 (2) Å; symmetry codes: (ii) 1 - *x*, 2 - *y*, -*z*; (v) 2 - *x*, 2 - *y*, 1 - *z*], C—H···N [*d*(C17···N1^vi^) = 3.435 (2) Å], C—H···C [*d*(C17···C2^vi^) = 3.603 (2) Å; symmetry code: (vi) 2 - *x*, 1 - *y*, -*z*] as well as C···Cl contacts [*d*(C8···Cl1^vii^) = 3.3888 (11) Å; symmetry code: (vii) 1 - *x*, 2 - *y*, 1 - *z*] (Fig. 3 and 4), forming an extended three-dimensional network.

### Experimental

Compound (I) was prepared, as a prospective ligand for syntheses of transition metal complexes, by the procedure described previously (Oh *et al.*, 1999). The resulting product was recrystallized from hot ethanol and crystals suitable for X-ray analysis were formed after several days of slow evaporation at room temperature. The crystals were characterized by elemental analysis, NMR spectroscopy and single-crystal X-ray analysis. ^1^H NMR (DMF-*d_7_*, TMS, 298 K, p.p.m.): 8.28 (s, 1H, C8H), 8.23 (t, 6.8, N6H, 1H), 7.23 (d, 8.2, C15H, 1H), 6.62 (d, 2.4, C12H, 1H), 6.49 (dd, 8.2, 2.4, C14H, 1H), 4.76 (sep, 6.8, C18H, 1H), 4.72 (d, 5.9, C9H, 2H), 3.89 (s, C16H, 3H), 3.80 (s, C17H, 3H), 1.58 (d, 6.8, C19H, C20H, 6H). ^13^C NMR (DMF-*d_7_*, TMS, 298 K, p.p.m.): *δ* 161.03 (C13), 158.99 (C11), 156.27 (C6), 154.01 (C2), 150.49 (C4), 139.96 (C8), 129.37 (C15), 120.32 (C10), 119.71 (C5), 104.90 (C14), 98.93 (C12), 55.88 (C16), 55.65 (C17), 47.90 (C18), 39.36 (C9), 22.42 (C19, C20). ^15^N NMR (DMF-*d_7_*, relative to DMF, 298 K, p.p.m.): *δ* 241.1 (N7), 228.5 (N1), 224.1 (N3), 179.6 (N9), 91.8 (N6). Analysis calculated for C_17_H_20_ClN_5_O_2_: C, 56.4; H, 5.6; N, 19.4. Found: C, 56.4; H, 5.9; N, 19.0%. Elemental analysis (C, H, N) was performed on a Thermo Scientific Flash 2000 CHNO-S Analyzer. The ^1^H, ^13^C and ^15^N NMR spectra of the DMF-*d_7_* solutions were obtained at 300 K on a Varian 400 spectrometer at 400.00 MHz, 100.58 MHz and 40.53 MHz respectively. ^1^H and ^13^C spectra were calibrated using tetramethylsilane (TMS) as a reference. The ^15^N NMR spectrum was measured relative to the DMF signals.

### Refinement

Non-hydrogen atoms were refined anisotropically and hydrogen atoms were located in difference maps and refined using the riding model with C—H = 0.95 (CH), C—H = 0.99 (CH_2_), C—H = 0.98 (CH_3_) Å, and N—H = 0.88 Å, with *U*_iso_(H) = 1.2*U*_eq_(CH, CH_2_, NH) and 1.5*U*_eq_(CH_3_).

### Crystal data

**Table:**

C_17_H_20_ClN_5_O_2_	*Z* = 2
*M**_r_* = 361.83	*F*(000) = 380
Triclinic, *P*1	*D*_x_ = 1.417 Mg m^−^^3^
Hall symbol: -P 1	Mo *K*α radiation, λ = 0.71073 Å
*a* = 7.8620 (2) Å	Cell parameters from 7872 reflections
*b* = 9.20164 (18) Å	θ = 3.0–31.9°
*c* = 13.3027 (3) Å	µ = 0.25 mm^−^^1^
α = 82.4472 (18)°	*T* = 100 K
β = 74.803 (2)°	Prism, colourless
γ = 66.012 (2)°	0.40 × 0.35 × 0.30 mm
*V* = 848.16 (3) Å^3^	

### Data collection

**Table:**

Agilent Xcalibur Sapphire2 diffractometer	2965 independent reflections
Radiation source: Enhance (Mo) X-ray Source	2704 reflections with *I* > 2σ(*I*)
Graphite monochromator	*R*_int_ = 0.009
Detector resolution: 8.3611 pixels mm^-1^	θ_max_ = 25.0°, θ_min_ = 3.0°
ω scans	*h* = −9→9
Absorption correction: multi-scan (*CrysAlis PRO*; Agilent, 2012)	*k* = −10→8
*T*_min_ = 0.908, *T*_max_ = 0.930	*l* = −15→15
7228 measured reflections	

### Refinement

**Table:**

Refinement on *F*^2^	Primary atom site location: structure-invariant direct methods
Least-squares matrix: full	Secondary atom site location: difference Fourier map
*R*[*F*^2^ > 2σ(*F*^2^)] = 0.029	Hydrogen site location: inferred from neighbouring sites
*wR*(*F*^2^) = 0.079	H-atom parameters constrained
*S* = 1.10	*w* = 1/[σ^2^(*F*_o_^2^) + (0.0452*P*)^2^ + 0.2812*P*] where *P* = (*F*_o_^2^ + 2*F*_c_^2^)/3
2965 reflections	(Δ/σ)_max_ = 0.001
230 parameters	Δρ_max_ = 0.28 e Å^−^^3^
0 restraints	Δρ_min_ = −0.19 e Å^−^^3^

### Special details

**Table:**

Geometry. All e.s.d.'s (except the e.s.d. in the dihedral angle between two l.s. planes) are estimated using the full covariance matrix. The cell e.s.d.'s are taken into account individually in the estimation of e.s.d.'s in distances, angles and torsion angles; correlations between e.s.d.'s in cell parameters are only used when they are defined by crystal symmetry. An approximate (isotropic) treatment of cell e.s.d.'s is used for estimating e.s.d.'s involving l.s. planes.
Refinement. Refinement of *F*^2^ against ALL reflections. The weighted *R*-factor *wR* and goodness of fit *S* are based on *F*^2^, conventional *R*-factors *R* are based on *F*, with *F* set to zero for negative *F*^2^. The threshold expression of *F*^2^ > σ(*F*^2^) is used only for calculating *R*-factors(gt) *etc*. and is not relevant to the choice of reflections for refinement. *R*-factors based on *F*^2^ are statistically about twice as large as those based on *F*, and *R*- factors based on ALL data will be even larger.

### Fractional atomic coordinates and isotropic or equivalent isotropic displacement parameters (Å^2^)

**Table:**

	*x*	*y*	*z*	*U*_iso_*/*U*_eq_	
Cl1	0.70269 (5)	1.09303 (4)	0.25218 (2)	0.02037 (12)	
O1	0.32700 (14)	0.87203 (11)	0.09283 (7)	0.0211 (2)	
O2	0.83383 (14)	0.47675 (11)	−0.12752 (7)	0.0219 (2)	
N1	0.58970 (15)	0.86255 (13)	0.31864 (8)	0.0154 (2)	
N3	0.75392 (16)	0.92987 (13)	0.42340 (8)	0.0158 (2)	
N6	0.48258 (16)	0.65791 (13)	0.35975 (8)	0.0157 (2)	
H6	0.4819	0.5716	0.3970	0.019*	
N7	0.63884 (16)	0.60705 (13)	0.55906 (8)	0.0164 (2)	
N9	0.77924 (15)	0.76630 (13)	0.58170 (8)	0.0149 (2)	
C2	0.67876 (18)	0.94292 (15)	0.34373 (10)	0.0151 (3)	
C4	0.72769 (18)	0.81144 (15)	0.48852 (10)	0.0143 (3)	
C5	0.64099 (18)	0.71309 (15)	0.47514 (10)	0.0145 (3)	
C6	0.56932 (18)	0.74185 (15)	0.38453 (10)	0.0142 (3)	
C8	0.72325 (19)	0.64331 (15)	0.62003 (10)	0.0170 (3)	
H8	0.7432	0.5891	0.6844	0.020*	
C9	0.38927 (19)	0.70512 (16)	0.27300 (10)	0.0157 (3)	
H9A	0.3342	0.8228	0.2670	0.019*	
H9B	0.2820	0.6692	0.2888	0.019*	
C10	0.51862 (18)	0.64023 (15)	0.16885 (10)	0.0148 (3)	
C11	0.47845 (18)	0.72793 (15)	0.07743 (10)	0.0156 (3)	
C12	0.58604 (19)	0.67038 (16)	−0.01979 (10)	0.0171 (3)	
H12	0.5559	0.7309	−0.0809	0.021*	
C13	0.73911 (19)	0.52284 (16)	−0.02749 (10)	0.0173 (3)	
C14	0.7841 (2)	0.43487 (16)	0.06153 (11)	0.0187 (3)	
H14	0.8896	0.3352	0.0566	0.022*	
C15	0.67172 (19)	0.49520 (15)	0.15851 (10)	0.0178 (3)	
H15	0.7016	0.4343	0.2196	0.021*	
C16	0.2745 (2)	0.96545 (17)	0.00354 (11)	0.0259 (3)	
H16A	0.3842	0.9866	−0.0399	0.039*	
H16B	0.1682	1.0665	0.0256	0.039*	
H16C	0.2350	0.9077	−0.0367	0.039*	
C17	0.9907 (2)	0.32597 (17)	−0.14131 (12)	0.0253 (3)	
H17A	1.0488	0.3093	−0.2158	0.038*	
H17B	0.9447	0.2416	−0.1120	0.038*	
H17C	1.0861	0.3235	−0.1056	0.038*	
C18	0.89162 (19)	0.83166 (15)	0.62220 (10)	0.0161 (3)	
H18	0.8547	0.9460	0.5990	0.019*	
C19	0.8456 (2)	0.82494 (17)	0.74010 (10)	0.0199 (3)	
H19A	0.8871	0.7136	0.7649	0.030*	
H19B	0.7073	0.8793	0.7669	0.030*	
H19C	0.9125	0.8776	0.7649	0.030*	
C20	1.1021 (2)	0.74454 (19)	0.57366 (12)	0.0267 (3)	
H20A	1.1445	0.6337	0.5995	0.040*	
H20B	1.1762	0.7962	0.5925	0.040*	
H20C	1.1219	0.7474	0.4977	0.040*	

### Atomic displacement parameters (Å^2^)

**Table:**

	*U*^11^	*U*^22^	*U*^33^	*U*^12^	*U*^13^	*U*^23^
Cl1	0.0287 (2)	0.02086 (19)	0.01726 (18)	−0.01511 (15)	−0.00878 (14)	0.00570 (13)
O1	0.0227 (5)	0.0188 (5)	0.0140 (5)	0.0003 (4)	−0.0057 (4)	0.0005 (4)
O2	0.0223 (5)	0.0198 (5)	0.0176 (5)	−0.0041 (4)	0.0001 (4)	−0.0037 (4)
N1	0.0163 (5)	0.0155 (6)	0.0139 (5)	−0.0065 (5)	−0.0019 (4)	−0.0005 (4)
N3	0.0176 (6)	0.0145 (5)	0.0153 (6)	−0.0066 (5)	−0.0037 (4)	0.0000 (4)
N6	0.0220 (6)	0.0169 (6)	0.0122 (5)	−0.0109 (5)	−0.0061 (5)	0.0024 (4)
N7	0.0208 (6)	0.0163 (6)	0.0131 (5)	−0.0085 (5)	−0.0038 (4)	0.0003 (4)
N9	0.0184 (6)	0.0153 (5)	0.0130 (5)	−0.0078 (5)	−0.0050 (4)	−0.0005 (4)
C2	0.0158 (6)	0.0134 (6)	0.0139 (6)	−0.0049 (5)	−0.0012 (5)	−0.0003 (5)
C4	0.0141 (6)	0.0132 (6)	0.0127 (6)	−0.0031 (5)	−0.0012 (5)	−0.0022 (5)
C5	0.0144 (6)	0.0145 (6)	0.0134 (6)	−0.0048 (5)	−0.0016 (5)	−0.0027 (5)
C6	0.0123 (6)	0.0133 (6)	0.0142 (6)	−0.0033 (5)	−0.0003 (5)	−0.0029 (5)
C8	0.0223 (7)	0.0160 (6)	0.0144 (6)	−0.0098 (6)	−0.0030 (5)	−0.0002 (5)
C9	0.0180 (6)	0.0178 (7)	0.0141 (6)	−0.0087 (5)	−0.0062 (5)	0.0012 (5)
C10	0.0178 (7)	0.0164 (7)	0.0153 (6)	−0.0108 (5)	−0.0054 (5)	0.0004 (5)
C11	0.0153 (6)	0.0150 (6)	0.0182 (7)	−0.0068 (5)	−0.0049 (5)	−0.0004 (5)
C12	0.0206 (7)	0.0174 (7)	0.0146 (6)	−0.0084 (6)	−0.0058 (5)	0.0024 (5)
C13	0.0178 (7)	0.0190 (7)	0.0173 (7)	−0.0098 (6)	−0.0022 (5)	−0.0032 (5)
C14	0.0195 (7)	0.0131 (6)	0.0228 (7)	−0.0045 (5)	−0.0068 (6)	−0.0012 (5)
C15	0.0231 (7)	0.0163 (7)	0.0179 (7)	−0.0098 (6)	−0.0094 (6)	0.0031 (5)
C16	0.0278 (8)	0.0227 (7)	0.0181 (7)	0.0001 (6)	−0.0084 (6)	0.0033 (6)
C17	0.0223 (7)	0.0218 (7)	0.0265 (8)	−0.0052 (6)	0.0006 (6)	−0.0071 (6)
C18	0.0198 (7)	0.0155 (6)	0.0168 (7)	−0.0091 (5)	−0.0063 (5)	−0.0012 (5)
C19	0.0240 (7)	0.0232 (7)	0.0162 (7)	−0.0114 (6)	−0.0065 (6)	−0.0013 (5)
C20	0.0212 (8)	0.0354 (9)	0.0258 (8)	−0.0124 (7)	−0.0010 (6)	−0.0129 (6)

### Geometric parameters (Å, º)

**Table:**

Cl1—C2	1.7537 (13)	C10—C11	1.4021 (18)
O1—C11	1.3703 (16)	C11—C12	1.3824 (19)
O1—C16	1.4213 (16)	C12—C13	1.3948 (19)
O2—C13	1.3700 (16)	C12—H12	0.9500
O2—C17	1.4270 (17)	C13—C14	1.3847 (19)
N1—C2	1.3256 (17)	C14—C15	1.3947 (19)
N1—C6	1.3574 (17)	C14—H14	0.9500
N3—C2	1.3130 (17)	C15—H15	0.9500
N3—C4	1.3496 (17)	C16—H16A	0.9800
N6—C6	1.3353 (17)	C16—H16B	0.9800
N6—C9	1.4532 (16)	C16—H16C	0.9800
N6—H6	0.8800	C17—H17A	0.9800
N7—C8	1.3201 (17)	C17—H17B	0.9800
N7—C5	1.3841 (17)	C17—H17C	0.9800
N9—C4	1.3655 (16)	C18—C19	1.5131 (18)
N9—C8	1.3676 (17)	C18—C20	1.5153 (19)
N9—C18	1.4831 (16)	C18—H18	1.0000
C4—C5	1.3866 (18)	C19—H19A	0.9800
C5—C6	1.4113 (18)	C19—H19B	0.9800
C8—H8	0.9500	C19—H19C	0.9800
C9—C10	1.5151 (18)	C20—H20A	0.9800
C9—H9A	0.9900	C20—H20B	0.9800
C9—H9B	0.9900	C20—H20C	0.9800
C10—C15	1.3799 (19)		
			
C11—O1—C16	117.98 (10)	C13—C12—H12	120.2
C13—O2—C17	117.58 (11)	O2—C13—C14	125.13 (12)
C2—N1—C6	117.18 (11)	O2—C13—C12	114.52 (12)
C2—N3—C4	109.33 (11)	C14—C13—C12	120.35 (12)
C6—N6—C9	121.82 (11)	C13—C14—C15	118.82 (12)
C6—N6—H6	119.1	C13—C14—H14	120.6
C9—N6—H6	119.1	C15—C14—H14	120.6
C8—N7—C5	103.89 (11)	C10—C15—C14	122.29 (12)
C4—N9—C8	105.86 (10)	C10—C15—H15	118.9
C4—N9—C18	124.22 (11)	C14—C15—H15	118.9
C8—N9—C18	129.61 (11)	O1—C16—H16A	109.5
N3—C2—N1	132.07 (12)	O1—C16—H16B	109.5
N3—C2—Cl1	114.30 (10)	H16A—C16—H16B	109.5
N1—C2—Cl1	113.62 (9)	O1—C16—H16C	109.5
N3—C4—N9	126.58 (12)	H16A—C16—H16C	109.5
N3—C4—C5	127.06 (12)	H16B—C16—H16C	109.5
N9—C4—C5	106.35 (11)	O2—C17—H17A	109.5
N7—C5—C4	110.24 (11)	O2—C17—H17B	109.5
N7—C5—C6	133.27 (12)	H17A—C17—H17B	109.5
C4—C5—C6	116.46 (12)	O2—C17—H17C	109.5
N6—C6—N1	118.06 (11)	H17A—C17—H17C	109.5
N6—C6—C5	124.07 (12)	H17B—C17—H17C	109.5
N1—C6—C5	117.87 (11)	N9—C18—C19	111.14 (10)
N7—C8—N9	113.66 (12)	N9—C18—C20	108.83 (10)
N7—C8—H8	123.2	C19—C18—C20	113.22 (12)
N9—C8—H8	123.2	N9—C18—H18	107.8
N6—C9—C10	114.74 (11)	C19—C18—H18	107.8
N6—C9—H9A	108.6	C20—C18—H18	107.8
C10—C9—H9A	108.6	C18—C19—H19A	109.5
N6—C9—H9B	108.6	C18—C19—H19B	109.5
C10—C9—H9B	108.6	H19A—C19—H19B	109.5
H9A—C9—H9B	107.6	C18—C19—H19C	109.5
C15—C10—C11	117.63 (12)	H19A—C19—H19C	109.5
C15—C10—C9	123.29 (12)	H19B—C19—H19C	109.5
C11—C10—C9	119.04 (11)	C18—C20—H20A	109.5
O1—C11—C12	123.76 (11)	C18—C20—H20B	109.5
O1—C11—C10	114.88 (11)	H20A—C20—H20B	109.5
C12—C11—C10	121.36 (12)	C18—C20—H20C	109.5
C11—C12—C13	119.54 (12)	H20A—C20—H20C	109.5
C11—C12—H12	120.2	H20B—C20—H20C	109.5

### Hydrogen-bond geometry (Å, º)

**Table:**

*D*—H···*A*	*D*—H	H···*A*	*D*···*A*	*D*—H···*A*
N6—H6···N7^i^	0.88	2.16	2.9465 (15)	148
C16—H16*C*···Cl1^ii^	0.98	2.78	3.4607 (15)	127
C19—H19*A*···O2^iii^	0.98	2.57	3.4709 (17)	154

Symmetry codes: (i) −*x*+1, −*y*+1, −*z*+1; (ii) −*x*+1, −*y*+2, −*z*; (iii) *x*, *y*, *z*+1.
